# Embryonic mouse medial neocortex as a model system for studying the radial glial scaffold in fetal human neocortex

**DOI:** 10.1007/s00702-022-02570-w

**Published:** 2022-11-30

**Authors:** Samir Vaid, Oskari Heikinheimo, Takashi Namba

**Affiliations:** 1 Department of Basic Neurosciences, University of Geneva, 1211 Geneva, Switzerland; 2 Department of Obstetrics and Gynecology, University of Helsinki and Helsinki University Hospital, P.O. 140, 00029 Helsinki, Finland; 3 Neuroscience Center, HiLIFE - Helsinki Institute of Life Science, University of Helsinki, P.O. 63, 00014 Helsinki, Finland

**Keywords:** Radial glial scaffold, Neuronal migration, Evolution, Mouse model

## Abstract

Neocortex is the evolutionarily newest region in the brain, and is a structure with diversified size and morphology among mammalian species. Humans have the biggest neocortex compared to the body size, and their neocortex has many foldings, that is, gyri and sulci. Despite the recent methodological advances in in vitro models such as cerebral organoids, mice have been continuously used as a model system for studying human neocortical development because of the accessibility and practicality of in vivo gene manipulation. The commonly studied neocortical region, the lateral neocortex, generally recapitulates the developmental process of the human neocortex, however, there are several important factors missing in the lateral neocortex. First, basal (outer) radial glia (bRG), which are the main cell type providing the radial scaffold to the migrating neurons in the fetal human neocortex, are very few in the mouse lateral neocortex, thus the radial glial scaffold is different from the fetal human neocortex. Second, as a consequence of the difference in the radial glial scaffold, migrating neurons might exhibit different migratory behavior and thus distribution. To overcome those problems, we propose the mouse medial neocortex, where we have earlier revealed an abundance of bRG similar to the fetal human neocortex, as an alternative model system. We found that similar to the fetal human neocortex, the radial glial scaffold, neuronal migration and neuronal distribution are tangentially scattered in the mouse medial neocortex. Taken together, the embryonic mouse medial neocortex could be a suitable and accessible in vivo model system to study human neocortical development and its pathogenesis.

**Supplementary Information:**

The online version contains supplementary material available at 10.1007/s00702-022-02570-w.

## Introduction

Brain has experienced enormous changes in its size and morphology during human evolution (Rakic 2009). The most significant changes are observed in the neocortex, the evolutionarily newest brain region which is responsible for higher order brain functions such as cognition (Rakic 2009). One of the prominent evolutionary changes of neocortex is folding, in the other words, gyri and sulci (Heuer et al. 2019; Namba et al. 2019). To accomplish a properly folded brain, neurons need to migrate in a coordinated manner, and disorganization of neuronal migration will result in neocortical malformations such as lissencephaly (Klingler et al. 2021).

Neurons are born in the germinal zones (ventricular zone, VZ; subventricular zone, SVZ) in the developing neocortex. The newly generated neurons then migrate toward the cortical plate (CP), which underlies the surface of the neocortex. Neuronal migration is known to be controlled by many cell-intrinsic and -extrinsic factors (Kawauchi and Hoshino 2008; Namba et al. 2015). Of several cell-extrinsic factors, radial glial scaffolds are thought to play a critical role in guiding neurons from the place of their birth to the final destination, the CP (Casingal et al. 2022). The radial glial scaffold is a structure transiently observed in the developing brain, and is mainly consisted of the basal process of the apical (ventricular) radial glia (aRG) and basal (outer) radial glia (bRG), which are located in the VZ and SVZ, respectively, and act as neural stem and progenitor cells (NPCs) (Casingal et al. 2022; Namba and Huttner 2017). The trajectory of the radial glial scaffold in the developing neocortex is known to be different between gyrencephalic (e.g., human, macaque and ferret) and lissencephalic species (e.g., mouse) (Gadisseux et al., 1992; Misson et al. 1991; Rakic 2003; Reillo et al. 2011). In the gyrencephalic species, the radial glial scaffold scatters more tangentially than in the lissencephalic species (Reillo et al. 2011). The tangentially scattered radial glial scaffold is thought to navigate the tangential dispersion of neurons, which finally leads to the folding of the neocortex.

Embryonic mouse neocortex has the advantage of accessibility and practicality of gene manipulation to examine the role of factors/genes influencing the radial glial scaffold and neuronal migration as a model system of human neocortex. However, there are two major disadvantages that might hinder the understanding of the human radial glial scaffold. First, the number of bRG is significantly smaller in the embryonic mouse neocortex than the fetal human neocortex (Kelava et al. 2012; Vaid et al. 2018; Wang et al. 2011), thus the majority of the radial glial scaffold consists of the aRG. Second, the radial glial scaffold is scattered less tangentially in the embryonic mouse neocortex, than the fetal human neocortex (Mission et al. 1991; Rakic 2003). We have previously shown that there is a region in the embryonic mouse neocortex where the proportion of bRG to other basal progenitors is similar to the fetal human neocortex at a specific developmental time point (Vaid et al. 2018). The region is the medial neocortex at E18.5. In the present study, we examined whether the structure of the radial glial scaffold in the embryonic mouse medial neocortex is similar to that in the fetal human neocortex to seek the possible utility of the embryonic mouse medial neocortex as a model system to study the human neocortical radial glial scaffold.

Here, we found that the directionality of the radial glial scaffold in the mouse medial neocortex exhibit a tangentially scattered pattern. Furthermore, neurons generated in the medial neocortex migrate tangentially to form a fan-shaped structure. Taken together, these results corroborate the idea that the mouse medial neocortex is a more suitable model system to study human neocortical development than the commonly used mouse lateral neocortex.

## Materials and methods

### Mice

C57BL/6J mice were used for all experiments using mouse tissue. Mice were maintained in pathogen-free conditions at the animal facility of the University of Helsinki, the University of Geneva, and the Max Planck Institute of Molecular Cell Biology and Genetics (MPI-CBG). All experiments were performed in accordance with Finnish, Swiss and German animal welfare legislation and were overseen by the Institutional Animal Welfare Officer. Necessary licenses were obtained from the regional ethical commission for animal experimentation in Helsinki (Aluehallintovirasto), in Geneve (Service de la consommation et des affaires vétérinaires), and in Dresden, Germany (Tierversuchskommission, Landesdirektion Dresden). Embryonic mouse brain tissues were dissected in PBS and immediately fixed with 4% PFA overnight at 4 °C, and then washed with PBS followed by 30% sucrose in PBS. The tissues were embedded in OCT compound (Sakura Finetek USA) and frozen using either liquid nitrogen or dry ice. The frozen samples were kept at − 80 °C for long-term storage.

### Human tissues

Fetal human brain tissue (GW19) was obtained from the Helsinki University Hospital, with approval by the ethics committee of the Hospital district of Helsinki and Uusimaa (HUS/1170/2021). Fetal human brain tissues were dissected in PBS and immediately fixed with 4% PFA overnight at 4 °C, and then washed with PBS followed by 30% sucrose in PBS. The tissues were embedded in OCT compound (Sakura Finetek USA) and frozen using liquid nitrogen. The frozen samples were kept at − 80 °C for long-term storage.

### Antibodies

Antibodies used in this study were as follows; anti-GFP (chicken IgY, GFP-1020, Aves Labs), anti-GLAST (guinea pig IgG, GLAST-GP-Af1000, Frontier institute, Japan), anti-nestin (mouse IgG, 611659, BD biosciences), anti-chicken IgY-Alexa Fluor 488 (donkey, 703–545-155, Jackson ImmunoResearch), anti-guinea pig IgG-Aexa Fluor 647 (donkey, 706–605-148, Jackson ImmunoResearch), anti-mouse IgG-Cy3 (donkey, 715–165-151, Jackson ImmunoResearch).

### In utero electroporation (IUE) of embryonic mouse neocortex

IUE was performed as previously described (Namba et al. 2020; Vaid et al. 2018). Briefly, pregnant mice with E13.5 embryos were anesthetized with isofluorane, followed by subcutaneous injection with the analgesic (0.1 ml, Metamizol, 200 mg/kg). In some cases Tamgesic (0.1 ml, Tamgesic, 200 mg/kg) was used as the analgesic and was given as one-shot 30 min before the surgery and second shot immediately after the surgery. Using a glass micropipette the embryos were injected intraventricularly with a solution containing 0.1% Fast Green (Sigma) in sterile 154 mM NaCl, 0.3 μg/μl of the pCAGGS-EGFP or pCAGGS-nlsRFP, followed by electroporation (24 V, five 50 ms pulses with 950 ms intervals). After surgery, when analgesic used during the surgery was Metamizol, mice received additional Metamizol for one day via drinking water (1.33 mg/ml). Mice were sacrificed either by cervical dislocation or by IP injection with pentobarbitol and embryos were harvested 72 h post-electroporation, and the embryonic brains were dissected and PFA-fixed for immunohistochemistry (see below) or processed for time-lapse imaging (see below).

### Immunohistochemistry

For nestin and GLAST immunofluorescence, 40 μm-thick coronal cryosections were stained as described previously (Vaid et al. 2018). Briefly, antigen retrieval was performed by incubating the sections, on the glass slide, in 0.01 M Na-citrate buffer for 60 min at 70 °C, followed by incubation for 20 min at room temperature. Sections were further permeabilized with 0.3% (wt/vol) Triton X-100 in PBS for 30 min and quenched with 2 mM glycine in PBS for 30 min, followed by blocking with a solution containing 0.2% (wt/vol) gelatin, 300 mM NaCl and 0.3% (wt/vol) Triton X-100 in PBS (blocking buffer). Primary antibodies were diluted in blocking buffer and sections incubated with primary antibodies overnight at 4 °C. After washing the section with PBS, the sections were incubated at room temperature with the appropriate secondary donkey antibodies and were counterstained with DAPI (Sigma, 1:1000). Sections were then mounted in Mowiol (Merck Biosciences).

### Time-lapse imaging

The slice culture was prepared as previously described (Namba et al. 2021). The electroporated brains with RFP were sectioned either manually or by tissue chopper into 250 μm slices. Then the slices were embedded in a collagen gel on a glass-bottom dish. The time-lapse images were taken by laser scanning confocal microscope LSM710 (Zeiss) with a Plan-apochromat 20 × objective. The images were taken every 15 min, for 30 h. During imaging, the slices were kept in an atmosphere of 5% CO_2_ + 40% O_2_ + 55% N_2_.

### Image acquisition and quantification

The fluorescent images were acquired using LSM710, LSM780 and LSM800, with 10x, 20x and 40x objectives. When the images were taken as tile scans, the images were stitched together using the ZEN software (Zeiss).

Quantifications were performed using the ZEN software and Fiji. Time-lapse imaging data were analyzed by TrackMate plugin of Fiji. The directionality of the nestin-positive fibers and tracks of RFP-positive cells was calculated by Fiji.

### Single cell RNA sequencing data analysis

Single cell RNA sequencing data processed in Vaid et al., (GSE120976) (Vaid et al. 2018) were analyzed and visualized using R 4.1.2 and R package Seurat v4.

### Statistical analysis

Data were analyzed with Excel (Microsoft, WA), Statcel3 (OMS, Japan), and MYSTAT (Systat Software, CA). Shapiro–Wilk normality test or the F test was used for testing normality or homoscedasticity of all samples, respectively. Two-tailed Student’s t-test was used for all two-group comparison. Results were interpreted as statistically significant when *p* < 0.05.

## Results

### Radial glial scaffold in the mouse medial neocortex at E18.5 exhibit a tangentially scattered pattern

**Fig. 1:**
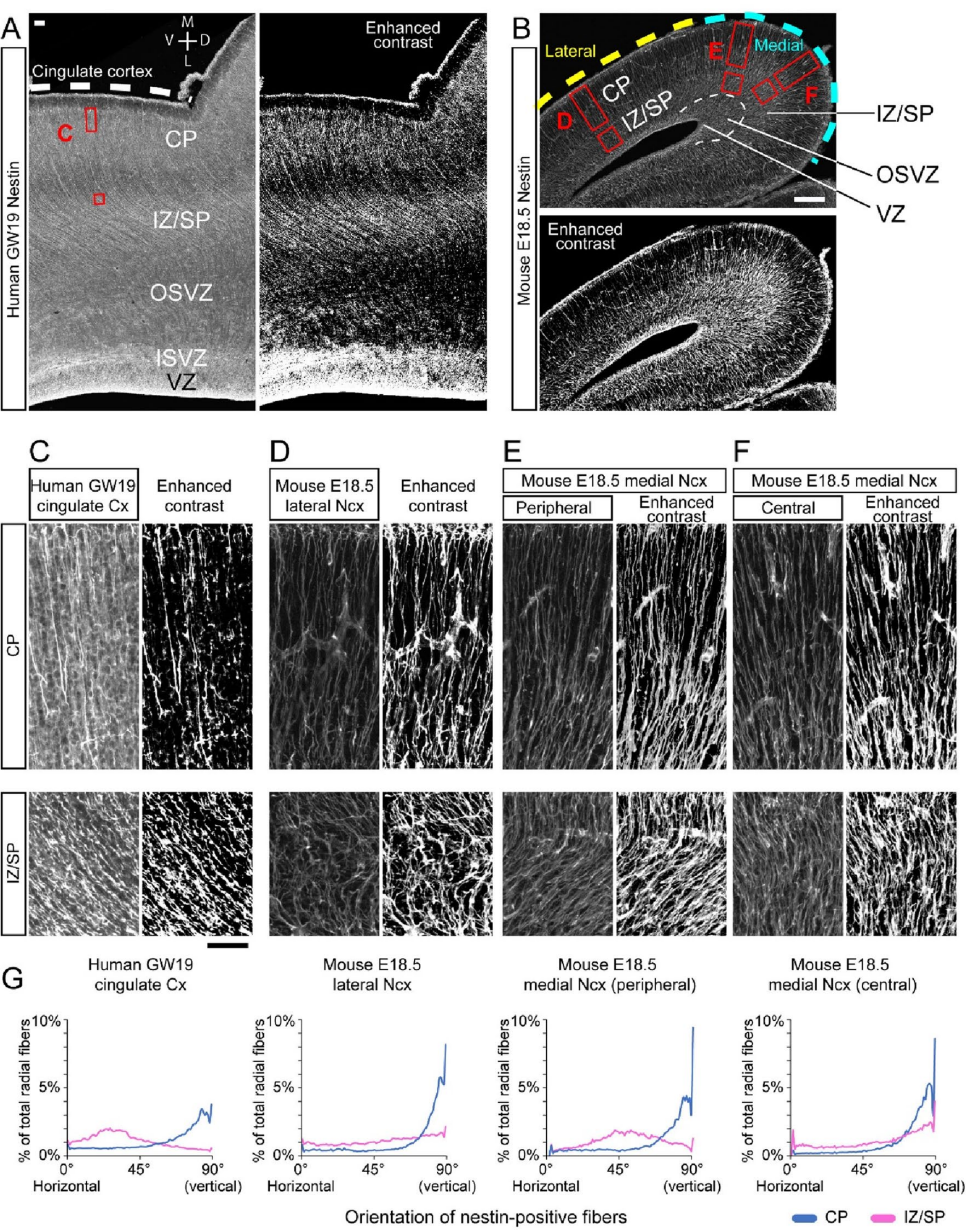
Radial glial scaffold in fetal human and embryonic mouse neocortex. (**A**) Nestin-positive fibers in human neocortex at GW19. The cingulate cortex is indicated by the dashed line. The regions indicated by the red boxes are shown at higher magnification in C. The right panel is an image with enhanced contrast. M: medial, L: lateral, D: dorsal, V: ventral. (**B**) Nestin-positive fibers in mouse neocortex at E18.5. The lateral and medial neocortex are indicated by the yellow and light blue dashed lines, respectively. The regions indicated by the red boxes are shown at higher magnification in D, E, and F. The dashed line in the medial neocortex indicates the border between IZ/SP and OSVZ-like structure. The bottom panel is an image with enhanced contrast. (**C–F**) Nestin-positive fibers in the CP (top panels) and the IZ/SP (bottom panels) of GW19 human (**C**), E18.5 mouse lateral neocortex (**D**), and E18.5 mouse medial neocortex (peripheral: **E**, central: **F**). The right panels are images with enhanced contrast. (**G**) Orientation of nestin-positive fibers in the CP (blue) and in the IZ/SP (magenta) of human cingulate cortex at GW19, mouse lateral and medial neocortex. The proportion of nestin-positive fibers at a specific angle to the total fibers is calculated every 1° from 0° (horizontal to the basal surface) to 90° (vertical: perpendicular to the basal surface). *n* = 4 for each region. *CP* cortical plate, *ISVZ* inner subventricular zone, *IZ/SP* intermediate zone/subplate, *OSVZ* outer subventricular zone, *VZ* ventricular zone. Scale bars: 200 μm in A and B, 50 μm in C applies to D-F

The radial glial scaffolds in the fetal human neocortex at gestational week (GW) 19 and embryonic mouse neocortex at E18.5 were visualized by nestin (Fig. 1) and GLAST (Supplementary Fig. 1) immunostaining. Since mouse medial neocortex will give rise to the cingulate and retrosplenial cortex, the human cingulate cortex was examined. In addition, since GW19 is the earliest stage at which cingulate cortex exhibit a sulcus (Chi et al. 1977; Namba et al. 2019), the cingulate cortex at GW19 is the best region to compare and analyze the ontogeny of the radial glial scaffolds in the gyrencephalic neocortex. The nestin-positive fibers in the CP of human neocortex were aligned largely parallel to each other and perpendicular to the surface of the neocortex (i.e., the basal surface) (Fig. 1A, C, G), whereas the fibers in the intermediate zone and subplate (IZ/SP) were aligned tangentially compared to the CP (Fig. 1A, C, G). Similar organization of the radial glial scaffold was also observed by the GLAST immunostaining (Supplementary Fig. 1). These results, especially the finding of tangential orientation in the regions apical to the CP, suggest that the radial glial scaffold in the human tissue exhibits a tangentially scattered pattern, which is consistent with the previous studies from macaque and ferret (Reillo et al. 2011).

In the mouse lateral neocortex at E18.5, the nestin-positive fibers in the CP were aligned perpendicular to the basal surface (Fig. 1B, D, G) as same as the fibers in the human fetal neocortex. In contrast, the nestin-positive fibers in the IZ/SP of the mouse lateral neocortex had a randomly but yet mostly vertical alignment (Fig. 1D, G), suggesting that the scaffold in the mouse lateral neocortex largely keeps radially oriented-manner with some randomness in the IZ/SP. The randomness observed in the IZ/SP might partially due to the branching of radial fibers (Takahashi et al. 1990). The nestin-positive fibers in the medial neocortex, however, exhibited distinct features. In the medial neocortex close to the lateral neocortex (we called as the peripheral medial neocortex), while the nestin-positive fibers aligned perpendicular to the basal surface in the CP as shown in the previous studies (Mission et al. 1991a; Mission et al. 1991b), the fibers in the IZ/SP bent toward the OSVZ-like structure in the medial neocortex (Fig. 1B, E, G). Therefore, similar to what is observed in the fetal human neocortex (Fig. 1C, G), the radial glial scaffold runs tangentially in the mouse medial neocortex at E18.5. Interestingly, the central part of the medial neocortex contained the nestin-positive fibers perpendicular to the basal surface all the way down to the OSVZ-like zone through the IZ/SP (Fig. 1B, F, G). Similar organization of the radial glial scaffold was also observed by the GLAST immunostaining (Supplementary Fig. 1). Therefore, the mouse medial neocortex possesses the fan-shaped radial glial scaffold, which is tangentially projected from the germinal zones toward the basal surface.

### Electroporated region is dispersed tangentially in the embryonic mouse medial neocortex

**Fig. 2:**
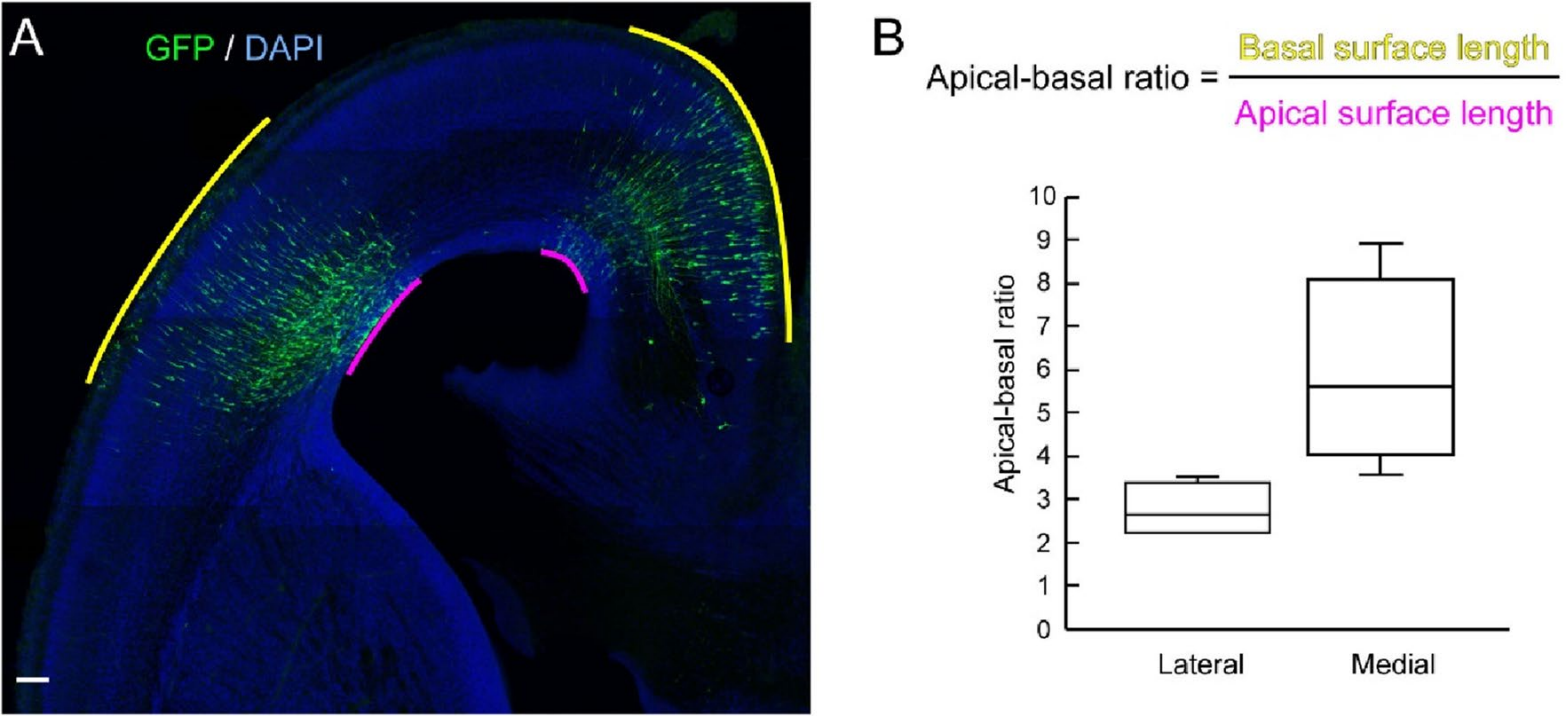
Electroporated region is dispersed tangentially in the embryonic mouse medial neocortex. (**A**) A representative image of electroporated regions at E18.5 mouse neocortex, which has been electroporated with GFP-expressing plasmid at E15.5. Yellow and magenta lines indicate the basal and apical surface of the electroporated regions. (**B**) Apical-basal ratio (a ratio of the basal surface length to the apical surface length) of the electroporated mouse lateral and medial neocortex at E18.5. Data within the boxes represent the 25–75% quartiles, and the line in the middle shows the median value. *n* = 4 embryos for each region

More tangentially scattered radial glial scaffold in the mouse medial neocortex than mouse lateral neocortex could affect the distribution of electroporated cells and their progenies. To test this hypothesis, embryonic mouse neocortex at E15.5 was electroporated with cytoplasmic GFP under the control of a ubiquitous promoter CAG to label both NPCs and neurons, and the electroporated area at E18.5 was analyzed to show to what extent the electroporated cells and their progenies are distributed. We compared the length of the apical surface and basal surface of the electroporated regions (we termed the ratio apical-basal ratio; Fig. 2A, B). If the ratio is close to 1, there is no tangential dispersion. While the apical-basal ratio in the lateral neocortex is approximately 2, the ratio in the medial neocortex is nearly 6 (Fig. 2B), showing that the progeny of the electroporated cells distribute tangentially three times more in the medial neocortex than the lateral neocortex.

### Neurons migrate tangentially in the embryonic mouse medial neocortex

**Fig. 3:**
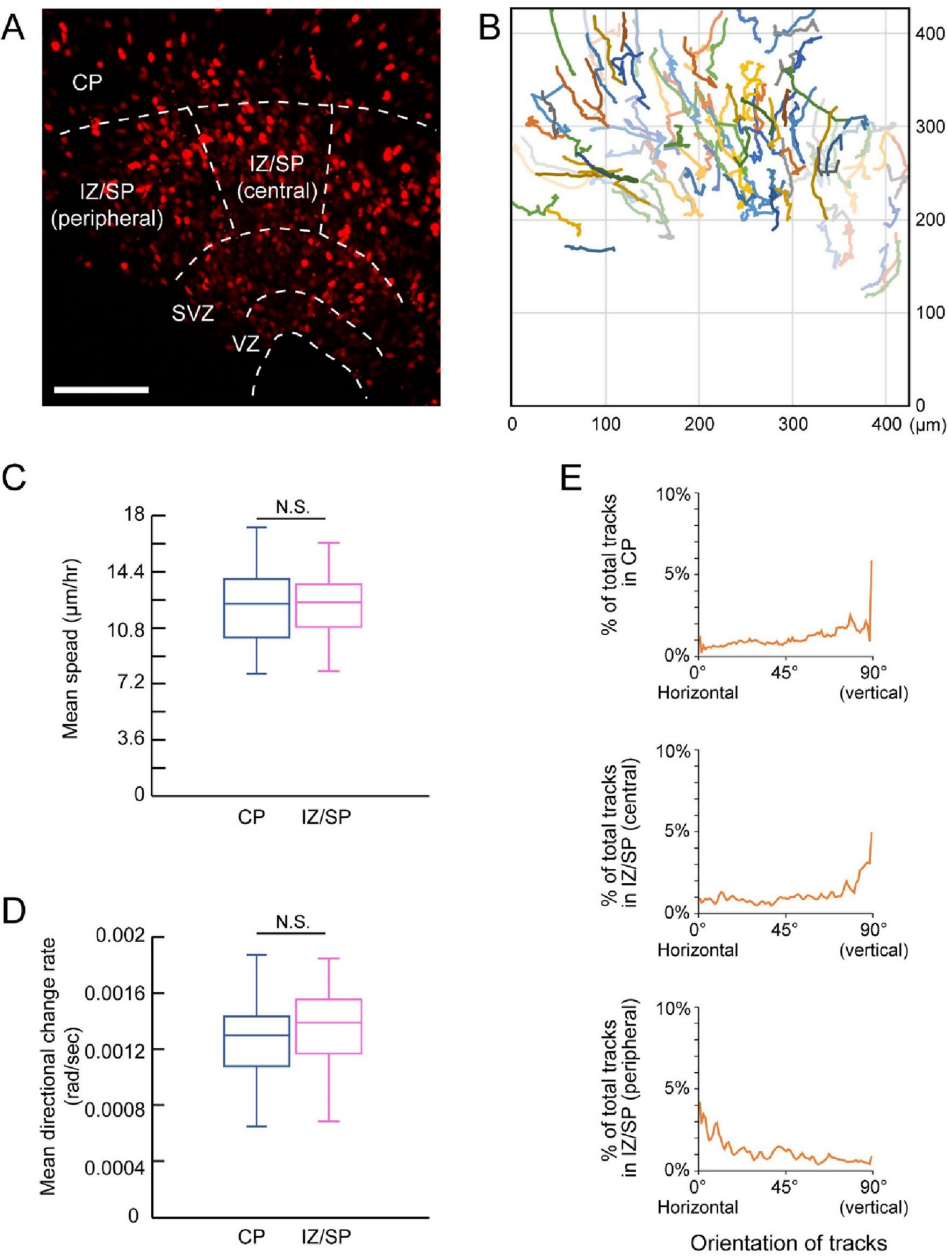
Neurons migrate more tangentially in the peripheral part of the IZ/SP in the mouse medial neocortex. (**A**) A representative image of electroporated regions at the beginning of time-lapse imaging of mouse neocortical slice, which has been electroporated with nslRFP-expressing plasmid at E15.5 and processed for imaging at E18.5. Dashed lines indicate borders of different regions labeled on the image. Scale bar: 100 μm. (**B**) Tracks of the RFP-positive cells in the CP and IZ/SP. (**C**, **D**) Mean speed (**C**) and mean directional change rate (**D**) of the RFP-positive cell migration in the CP and in the IZ/SP. Data within the boxes represent the 25–75% quartiles, and the line in the middle shows the median value. Total 58 cells and 63 cells were analyzed for the CP and IZ/SP, respectively. (**E**) Orientation of the tracks of RFP-positive cell migration in the CP, and in the central and peripheral part of IZ/SP. The proportion of the tracks at a specific angle to the total tracks is calculated every 1° from 0° (horizontal to the basal surface) to 90° (vertical: perpendicular to the basal surface). Total 121 cells from 3 experiments were analyzed

The tangential dispersion of the electroporated region in the mouse medial neocortex rises the possibility that the newly generated neurons migrate tangentially in the medial neocortex. There are three major migratory modes of neurons in the developing neocortex (Kawauchi and Hoshino 2008; Namba et al. 2015). Locomotion and somal translocation are the characteristic radial glial scaffold-dependent migration of neurons, which allow neurons to migrate long distances (Kawauchi and Hoshino 2008; Namba et al. 2015). The other mode is so-called multipolar migration, which is the radial glial scaffold-independent and typically exhibits slow speed and thus short distance travel in a given period (Kawauchi and Hoshino 2008; Namba et al. 2015; Tabata and Nakajima 2003). We electroporated the medial neocortex of mouse embryos at E15.5 with nuclear-localized RFP under the control of the CAG promoter, and then the neocortical tissues were processed for slice culture and time-lapse imaging (Fig. 3A). RFP-positive cells in the CP and IZ/SP without cell division are considered neurons. To focus on the radial glial scaffold-dependent neuronal migration, this study only analyzed the neurons that migrate more than 40 μm in direct distance between the start and end of the track during the imaging period (13 h; Fig. 3B). There are no differences in the speed and directional change rate of RFP-positive neurons in the CP and IZ/SP (Fig. 3C, D). However, the RFP-positive neurons in the peripheral IZ/SP migrate more tangentially than the neurons in the CP or in the central IZ/SP (Fig. 3B, E). These results suggest that the neurons in the medial neocortex migrate tangentially, prominently in the peripheral part, to accomplish the tangential distribution of neurons.

## Discussion

To analyze human neocortical development, there are currently several options. Each option has advantages and disadvantages (Table 1). For example, ferret, a gyrencephalic carnivore, is used for studying neocortical expansion and folding (Kalebic et al. 2018; Kawasaki et al. 2013; Reillo and Borrell 2012), however, the accessibility is limited due to the high cost of the animals and their housing. In addition, the gene knockout ferrets have been reported (Johnson et al. 2018) but have not been widely used so far. Cerebral organoids generated from human iPSCs are now widely used because of the established methods for genome editing in iPSCs and the fewer ethical problems (Eiraku et al. 2008; Lancaster et al. 2017). However, it is still on the way to the organoids with the quality comparable to the fetal human neocortical tissue in vivo (Pollen et al. 2019), especially the abundance of bRG, neuronal layer formation and gyrification.

**Table 1:** Summary of advantages and disadvantages of each model systems

	Human fetal neocortex	Human cerebral organoids	Ferret embryonic neocortex	Mouse embryonic lateral neocortex	Mouse embryonic medial neocortex
Gyrification	Yes	Needs improvement	Yes	No	No
bRG abundance	High	Needs improvement	High	Low	High
Fan-shaped RG scaffold	Yes	No data	Yes	No	Yes
Tangential migration of pyramidal neurons	Yes	No data	Yes	Less	Yes
Highly annotated genome	Yes	Yes	Less annotated	Yes	Yes
Gene KO/manipulation	Yes (ex vivo electroporation)	Yes	Yes (mainly in utero electroporation)	Yes (KO mice and in utero electroporation)	Yes (KO mice and in utero electroporation)
RNA sequencing data	Many	Many	No	Many	Not many
Accessibility	Low	High	Low-mid	High	High

Mice have decades of history as an in vivo experimental model system which allows us to analyze neocortical development to cognitive functions associated with the neocortex upon gene manipulations. In many studies, the lateral neocortex has been chosen as a region of interest, therefore a large amount of accumulated knowledge on brain development is available (Coquand et al. 2021; Kawauchi et al. 2010; Nakagawa et al. 2019; Namba et al. 2014; Shitamukai et al. 2011). However, there are considerable differences in the developmental process between the mouse lateral neocortex and human neocortex. First, the abundance of bRG is significantly lower in the embryonic mouse lateral neocortex (approximately 10% of total basal progenitors) compared to the fetal human neocortex (approximately 50% of total basal progenitors) (Kelava et al., 2012; Vaid et al. 2018; Wang et al. 2011). Second, less tangential dispersion of the radial glial scaffold (Gadisseux et al. 1992; Rakic, 2003), thus neurons (Cortay et al., 2020; Gertz and Kriegstein 2015; Hara et al. 2016; Martinez-Martinez et al. 2018), are observed throughout the embryonic period. It is important to consider these features when we study neocortical development using mice as a model.

We have previously proposed that the mouse medial neocortex at E18.5 is a better suited model system for studying human neocortex, since the mouse medial neocortex contains an abundance of bRG (approximately 50% of total basal progenitors) which is comparable to the fetal human neocortex (Vaid et al. 2018). Since then, several groups have started to use the mouse medial neocortex as a model system of human neocortical bRG (Bertacchi et al. 2020; Kerimoglu et al. 2021; Pinson et al. 2022; Wang et al. 2022). In the present study, we aim to further expand the utility of the mouse medial neocortex to studying radial glial scaffold which regulates neuronal migration. Since newly generated neurons are known to migrate more tangentially in the IZ/SP of gyrencephalic species, and reduction of tangential migration has been shown to affect the gyrus volume (Reillo et al. 2010), tangentially scattered radial glial scaffold and neuronal migration are important features to be addressed in the context of examining cortical malformation-associated genes in mice. Interestingly, the mouse medial neocortex is prone to develop folding upon genetical manipulations (Wang et al. 2016, 2022), suggesting that the tangentially scattered radial glial scaffold and neuronal migration described in the present study could be involved in the ectopic gyrification in the mouse medial neocortex.

The tangential neuronal migration in gyrencephalic neocortex is known to be regulated by cell-to-cell interaction (Del Toro et al. 2017). Expression of the fibronectin leucine rich-repeat transmembrane protein (FLRT), a cell adhesion molecule, is higher in the embryonic mouse neocortex than the fetal human neocortex. The higher FLRTs expression in the mouse lateral neocortex is required for the radial migration of neurons, and their reduction induces tangential neuronal dispersion, thus neocortical folding. Interestingly, *Flrt1*, *Flrt2* and *Flrt3* gene expression has been shown to be highest in the lateral neocortex, and lower in the medial neocortex of embryonic mouse (Del Toro et al. 2017; Yamagishi et al. 2011). Consistent with this previous study, only a minor population of neurons in the mouse medial neocortex express *Flrt1* (Supplementary Fig. 2), suggesting a possibility that the lower expression of FLRTs in the mouse medial neocortex enable the neurons migrate tangentially along the fan-shaped radial glial scaffold.

Unfortunately, mice are not the perfect model system to study human neocortical development, and never will be, because there are significant contributions of human- and primate-specific genes in human neocortical development (Fiddes et al. 2018; Florio et al. 2015, 2018; Liu et al. 2017; Namba et al. 2020; Pinson et al. 2022; Suzuki 2020; Suzuki et al. 2018; Vaid and Huttner 2020; Van Heurck et al. 2022). However, using the mouse medial neocortex as a model system appears to be a good compromise in terms of bRG abundance, and the tangentially scattered radial glial scaffold and neuronal migration. Therefore, the mouse medial neocortex is a “poor man’s Porsche”, an alternative, cheaper and more accessible way to study human neocortical development and its pathogenesis.

## Supplementary Information

Below is the link to the electronic supplementary material.

**Supplementary Figure 1. GLAST-positive radial glial scaffold in fetal human and embryonic mouse neocortex** (**A**) GLAST-positive fibers in human neocortex at GW19. The cingulate cortex is indicated by the dashed line. The regions indicated by the red boxes are shown at higher magnification in C. The right panel is an image with enhanced contrast. M: medial, L: lateral, D: dorsal, V: ventral. (**B**) GLAST-positive fibers in mouse neocortex at E18.5. The lateral and medial neocortex are indicated by the yellow and light blue dashed lines, respectively. The regions indicated by the red boxes are shown at higher magnification in D, E, and F. The dashed line in the medial neocortex indicates the border between IZ/SP and OSVZ-like structure. The bottom panel is an image with enhanced contrast. (**C-F**) GLAST-positive fibers in the CP (top panels) and the IZ/SP (bottom panels) of GW19 human (**C**), E18.5 mouse lateral neocortex (**D**), and E18.5 mouse medial neocortex (peripheral: **E**, central: **F**). The right panels are images with enhanced contrast. CP: cortical plate, ISVZ: inner subventricular zone, IZ/SP: intermediate zone/subplate, OSVZ: outer subventricular zone, VZ: ventricular zone. Scale bars: 200μm in **A** and **B**, 50μm in **C** applies to **D-F**.

## Data Availability

Single cell RNA sequencing data are publicly available (GSE120976). The datasets generated and/or analysed during the current study are available from the corresponding author on reasonable request.
