# Supplementary material for: Embryonic mouse medial neocortex as a model system for studying the radial glial scaffold in fetal human neocortex
Source: J Neural Transm (Vienna). 2022 Nov 30;130(3):185–94. doi: 10.1007/s00702-022-02570-w (PMC10033555; doi:10.1007/s00702-022-02570-w)
Supplement: Supplementary file 1 — Supplementary Figure 1. GLAST-positive radial glial scaffold in fetal human and embryonic mouse neocortex (A) GLAST-positive fibers in human neocortex at GW19. The cingulate cortex is indicated by the dashed line. The regions indicated by the red boxes are shown at higher magnification in C. The right panel is an image with enhanced contrast. M: medial, L: lateral, D: dorsal, V: ventral. (B) GLAST-positive fibers in mouse neocortex at E18.5. The lateral and medial neocortex are indicated by the yellow and light blue dashed lines, respectively. The regions indicated by the red boxes are shown at higher magnification in D, E, and F. The dashed line in the medial neocortex indicates the border between IZ/SP and OSVZ-like structure. The bottom panel is an image with enhanced contrast. (C-F) GLAST-positive fibers in the CP (top panels) and the IZ/SP (bottom panels) of GW19 human (C), E18.5 mouse lateral neocortex (D), and E18.5 mouse medial neocortex (peripheral: E, central: F). The right panels are images with enhanced contrast. CP: cortical plate, ISVZ: inner subventricular zone, IZ/SP: intermediate zone/subplate, OSVZ: outer subventricular zone, VZ: ventricular zone. Scale bars: 200μm in A and B, 50μm in C applies to D-F. [file 702_2022_2570_MOESM1_ESM.docx]

**Supplementary Figures**


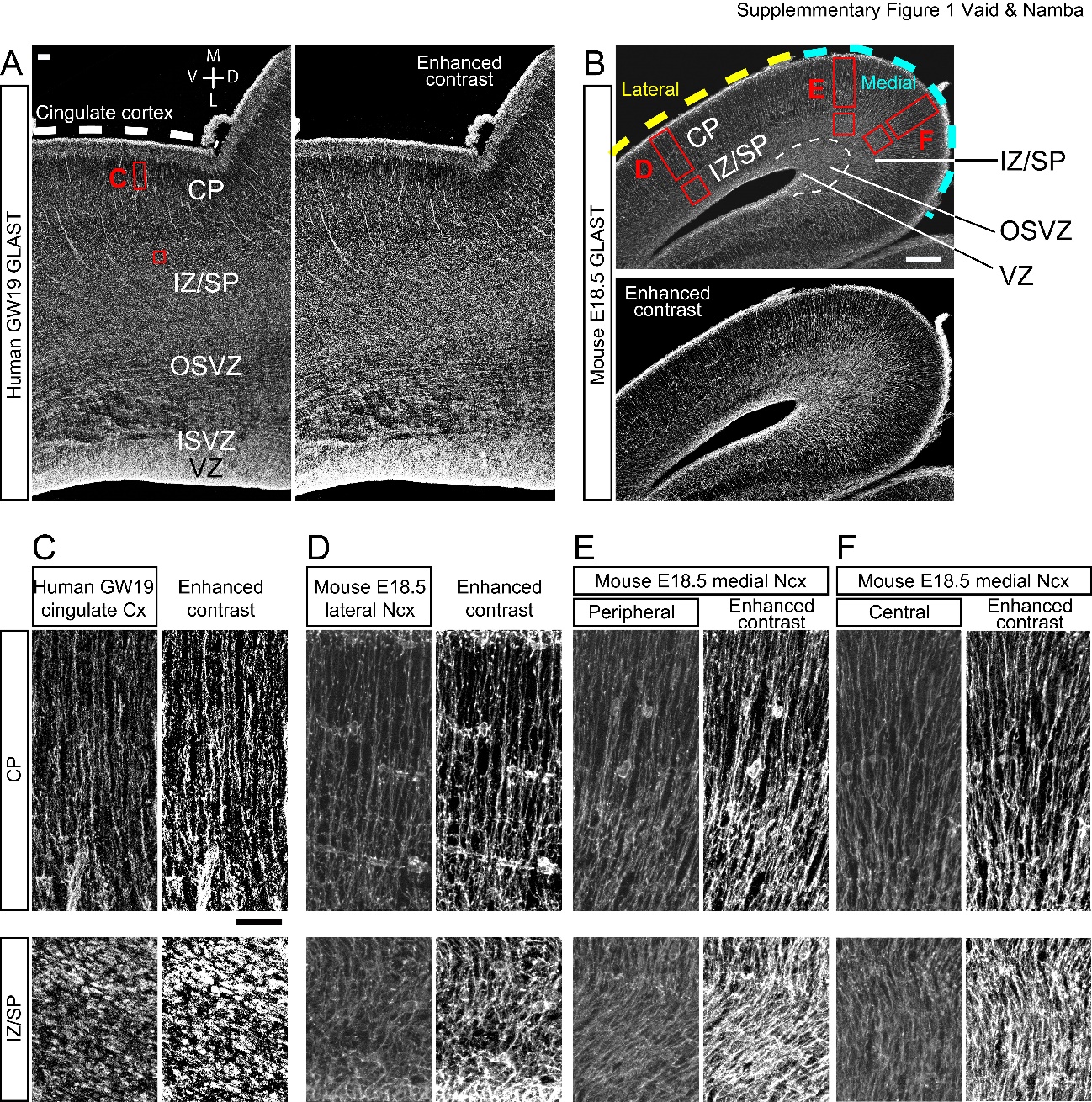


**Supplementary Figure 1.** **GLAST-positive radial glial scaffold in fetal human and embryonic mouse neocortex**

(A) GLAST-positive fibers in human neocortex at GW19. The cingulate cortex is indicated by the dashed line. The regions indicated by the red boxes are shown at higher magnification in C. The right panel is an image with enhanced contrast. M: medial, L: lateral, D: dorsal, V: ventral.

(B) GLAST-positive fibers in mouse neocortex at E18.5. The lateral and medial neocortex are indicated by the yellow and light blue dashed lines, respectively. The regions indicated by the red boxes are shown at higher magnification in D, E, and F. The dashed line in the medial neocortex indicates the border between IZ/SP and OSVZ-like structure. The bottom panel is an image with enhanced contrast.

(C-F) GLAST-positive fibers in the CP (top panels) and the IZ/SP (bottom panels) of GW19 human (C), E18.5 mouse lateral neocortex (D), and E18.5 mouse medial neocortex (peripheral: E, central: F). The right panels are images with enhanced contrast.

CP: cortical plate, Cx: cortex, ISVZ: inner subventricular zone, IZ/SP: intermediate zone/subplate, Ncx: neocortex, OSVZ: outer subventricular zone, VZ: ventricular zone. Scale bars: 200μm in A and B, 50μm in C applies to D-F.


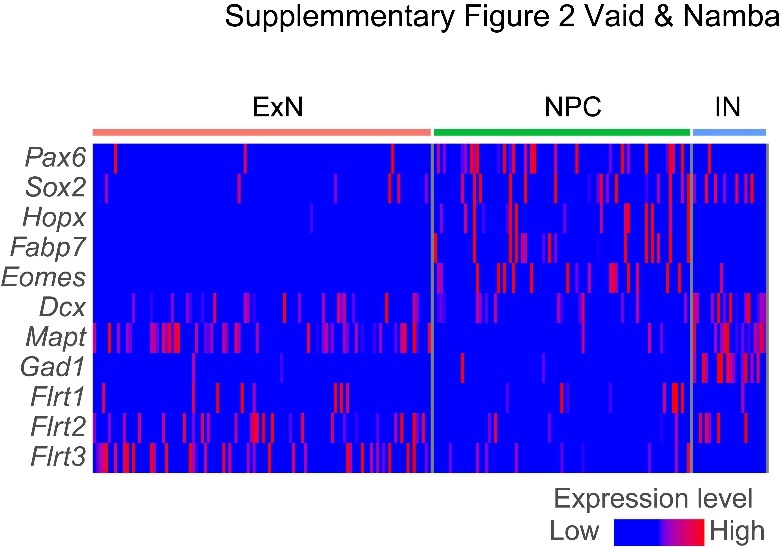


**Supplementary Figure 2.** ***Flrt1*, *Flrt2* and *Flrt3* expression in the mouse medial neocortex**

Single cell RNA sequencing analysis of mouse medial neocortex at E18.5. The data are obtained from GSE120976 and re-analyzed. ExN: excitatory neurons, NPC: neural progenitor cells including AP and BP, IN: inhibitory neurons. Cells are in columns, genes are in rows.
